# Supplementary material for: Engineering of Genetically Encoded Bright Near-Infrared Fluorescent Voltage Indicator
Source: Int J Mol Sci. 2025 Feb 8;26(4):1442. doi: 10.3390/ijms26041442 (PMC11855178; doi:10.3390/ijms26041442)
Supplement: Supplementary file 1 [file ijms-26-01442-s001.zip › ijms-3446473-supplementary.pdf]

Engineering of Genetically Encoded Bright Near-Infrared Fluorescent Voltage Indicator

Supplementary Figures

Figure S1. Alignment of amino acid sequences of Archaerhodopsin-3 (Arch), Archer1, QuasAr1, QuasAr2, Archon1, Archon3, monArch, QuasAr6a and QuasAr6b.

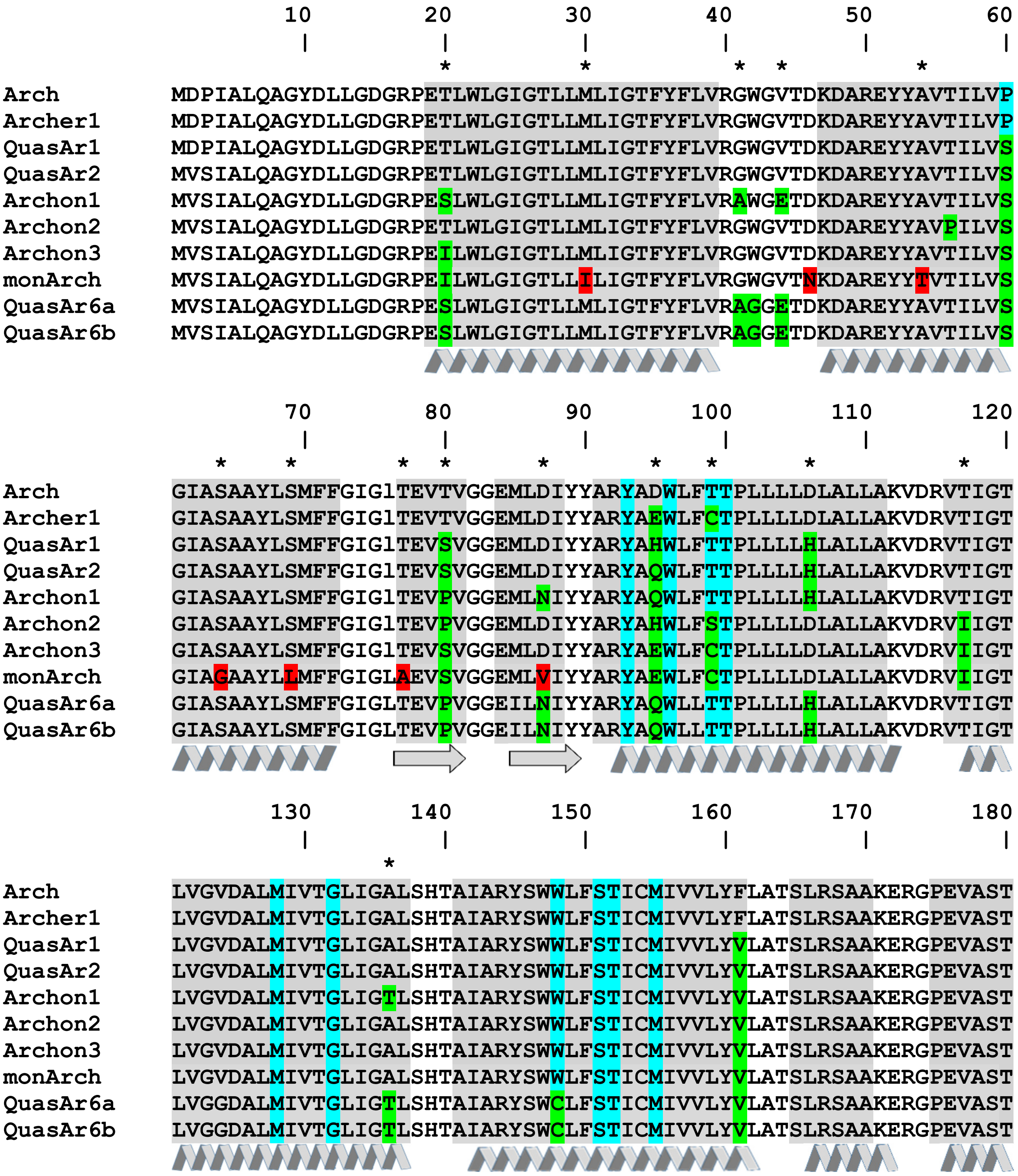

```
                        190       200       210       220       230       240
                          |         |         |         |         |         |
                                                                *
Arch         FNTLTALVLVLWTAYPILWIIGTEGAGVVGLGIETLLFMVLDVTAKVGFGFILLRSRAIL
Archer1      FNTLTALVLVLWTAYPILWIIGTEGAGVVGLGIETLLFMVLDVTAKVGFGFILLRSRAIL
QuasAr1      FNTLTALVLVLWTAYPILWIIGTEGAGVVGLGIETLLFMVLDVTAKVGFGFILLRSRAIL
QuasAr2      FNTLTALVLVLWTAYPILWIIGTEGAGVVGLGIETLLFMVLDVTAKVGFGFILLRSRAIL
Archon1      FNILTALVLVLWTAYPIIWIIGTEGAGVVGLGIETLLFMVLDVTAKVGFGFILLRSRAIL
Archon2      FNILTALVLVLWTAYPIIWIIGTEGAGVVGLGIETLLFMVLDVTCKVGFGFILLRSRAIL
Archon3      FNILTALVLVLWTAYPIIWIIGTEGAGVVGLGIETLLFMVLDVTGKVGFGFILLRSRAIL
monArch      FNILTALVLVLWTAYPIIWIIGTEGAGVVGLGIETLLFMVLDVTGKVGFGFILLRSRAIL
QuasAr6a     FNILTALVLVLWTAYPIIWIIGTEGAGVVGLGIETLLFMVLDVTAKVGFGFILLRSRSIL
QuasAr6b     FNILTALVLVLWTAYPIIWIIGTEGAGVVGLGIETLLFMVLDVTAKVGFGFILLRSIAIL
```

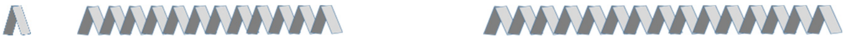

```
                      250
                        |
              **
Arch          GDTEAPEPSAGADVSAAD
Archer1       GDTEAPEPSAGAD
QuasAr1       GDTEAPEPSAGAD
QuasAr2       GDTEAPEPSAGAD
Archon1       QDTEAPEPSAGAD
Archon2       GDTEAPEPSAGAD
Archon3       GDTEAPEPSAGAD
monArch       GVTEAPEPSAGAD
QuasAr6a      QDTEAPEPSAGAD
QuasAr6b      QDTEAPEPSAGAD
```

Amino acid numbering follows that of Arch. The chromophore-surrounding residues (within 4.0 Å) are highlighted in cyan. Residues that are different from Arch are highlighted in green. Mutations in monArch that are different from Archon3 are highlighted in red. Residues in monArch that are different from Archon1 are marked with asterisks. The β-sheet-forming regions and α-helixes are shaded and denoted with arrows and ribbons, respectively.

Figure S2. Predicted structure of Arch-derived GEVIs.

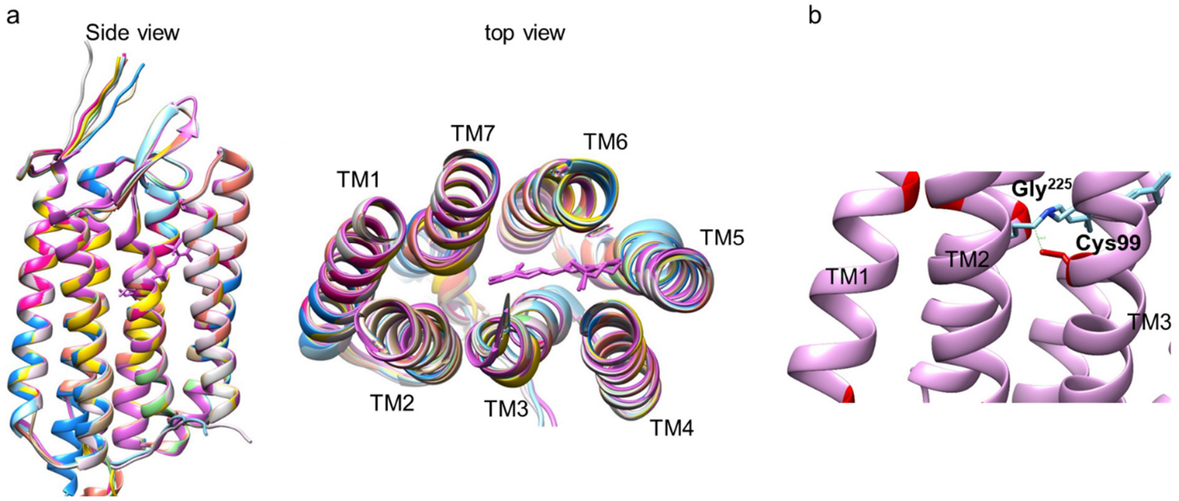

a, Three-dimensional structure of Arch-derived GEVIs (Archer1, QuasAr1, QuasAr2, Archon1, Archon2, Archon3, QuasAr6a, QuasAr6b, monArch) were predicted using AlphaFold3. The Arch structure (PDB: 6GUX) was shown in magenta. **b,** Two residues (Cys^{99} and Gly^{225}, in red) in monArch in close proximity to the chromophore (in cyan) were shown.

Figure S3. Expression of soma-monArch in mouse brain slices.

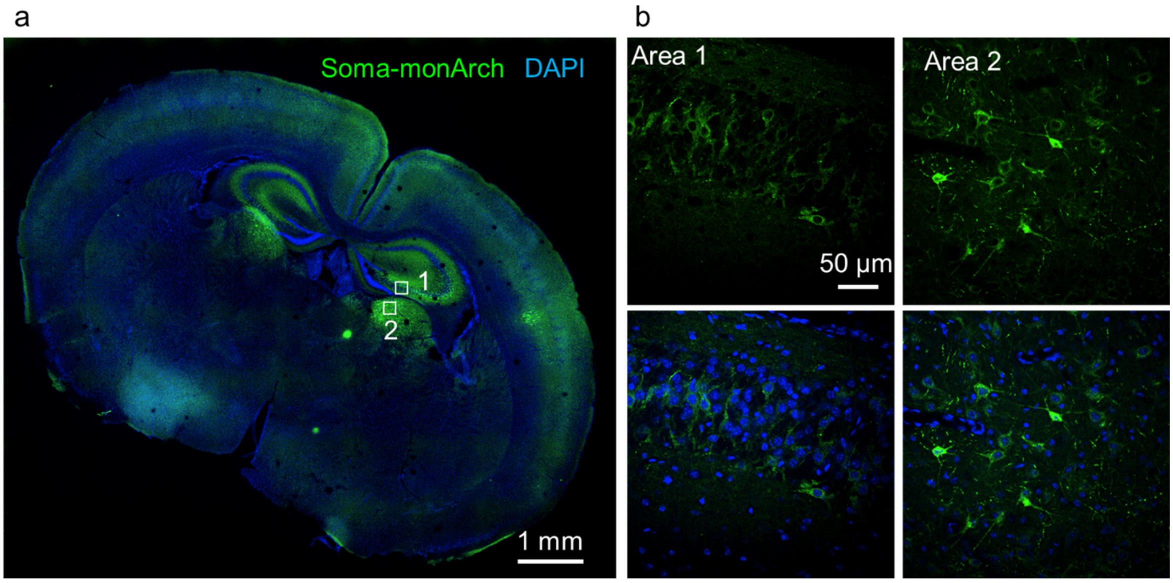

Confocal images showing the expression of Soma-monArch in mouse brain. rAAV-CaMKII-monArch-KGC-EGFP-**Kv2.1**$_{\mathbf{motif}}$-ER2 (soma-monArch) was injected into P0 mouse brain. After four week's expression, the mice were sacrificed for histology. **a**. Whole brain overview of soma-monArch expression. **b**, higher magnification single-plane images showing the expression of soma-monArch in the two areas (hippocampus and thalamic nucleus) of the mouse brain indicated in the panel a.
